# Supplementary material for: Brief communication: Plasma cortisol concentration is affected by lactation, but not intra-nasal oxytocin treatment, in beef cows
Source: PLoS One. 2021 Jul 30;16(7):e0249323. doi: 10.1371/journal.pone.0249323 (PMC8323922; doi:10.1371/journal.pone.0249323)
Supplement: Attachment — Submitted filename: PLOS Response to Reviewers comments Lact.docx [file pone.0249323.s002.docx]

**Brief communication: Plasma cortisol concentration is affected by lactation, but not intra-nasal oxytocin treatment, in beef cows.**

**Response to Reviewers comments.**

Reviewer #1: Authors should consider modifying Figure 1 so it contains the circulating cortisol profiles for all 4 treatments. No oxytocin treatment by lactation effects occurred but there still is merit in showing all 4 profiles. This information is, after all, what the study was designed to test.

AU Response: We thank the reviewer for their suggestion and have included an additional figure that contains the cortisol concentration over time for all four treatments.

Reviewer #2: Line 28: 0 minutes relative to what? It is unclear what the starting point was. It is recommended that the timing be changed where time 0 is the time of the oxytocin challenge (i.e., -60, -30, -15, 0, 2, 4, 6, etc)

AU Response: The text and figures have been amended to reflect reviewer #2 comments for the X axis with the oxytocin or saline treatment occurring at time 0-minute.

Line 28: Why were cannulation and treatment/sample collection performed on the same day? Cannulation is stressful and can cause an increase in cortisol.

AU Response: The reviewer is correct that cannulation can be stressful and can cause an increase in cortisol in some animals. The response of the animal to handling and husbandry procedures is, however, dependent upon the experience of the animal and the herd culture. For example, Hopster et al. (1999) demonstrated cows accustomed to handling and head restraint in a self-locking feed gate enabled baseline cortisol concentration to be obtained via jugular puncture within 1 minute of approaching the cow (Hopster et al. 1999. Effects of repeated jugular puncture on plasma cortisol concentrations in loose housed dairy cows. J. Anim. Sci. 77:708-714). The cows in the present study have been extensively handled throughout their lives by experienced cattle handlers that adopt low stress handling procedures. Furthermore, reproductive, and physiological studies requiring serial blood sampling and handling is common for the beef cow herd at The Ohio State University. Our experience, with these cattle is that most of the cows in the herd will respond to cannulation with a minor increase in cortisol and they resume baseline concentration of cortisol within 2 hours. In addition, we use a sub-cutaneous anesthetic before we began the cannulation process to avoid pain in the animal. Moreover, despite the cannulation procedure the lactating cows demonstrated reduced plasma cortisol concentration when compared with non-lactating cows.

Line 28: Based on what is described, were cannulations/treatments/sample collection staggered throughout the day? Is there a concern of any diurnal effects on cortisol production?

AU Response: Diurnal variation in cortisol concentration does exist in cows. However, animals from each treatment were randomly assigned to a time either in the morning or in the afternoon. Where possible all treatments were represented at each time (am or pm) and therefore any diurnal effects on cortisol concentration in the cows would have been minimized.

Line 57: exposed to the stressor for how long?

AU Response: The psycho-social stress imposed by Ralph and Tillbrook was for a period of 4 hours. We have amended the manuscript to include the time of exposure to the stressor in the sentence.

Line 62: oxytocin administration independent of stressor

AU Response: We have amended the sentence to include the reviewer’s suggestion

Line 77: Time of day is known to affect production and secretion of many hormones, including cortisol. Was time of day taken into consideration in the model?

AU Response: Yes the time of day and day of procedure was included in the model but removed because of a lack of effect. We inadvertently did not include this information in the original manuscript. We have amended the manuscript to state “Day and time of day (AM or PM) were included in the model as fixed effects; however, no effect of either day or time of day was detected and these variables were removed from the model.”

Line 79: Was calf present only for lactating dams? If so, could this be a confiding factor?

AU Response: Yes, only the lactating cows had their calf present in front of them. We see a lactating beef cow and her calf as one unit. We have amended the manuscript to reflect that the experimental unit used and therefore the interpretation of the data were for “lactating cows with access to their calves” instead of lactation alone.

Line 82: Was this 2 hours on average or was blood sampling staggered based on catherization.

AU Response: Sampling was based on time of catheterization. We have amended the manuscript to state, “Blood sampling was staggered based on the time of catheterization for each animal.”

Line 84: How as the baseline blood sample collected?

AU Response: Baseline blood samples were collected with a 10mL syringe via the jugular catheter.

Line 91: Please describe extension sets.

AU Response: A description of the extension set has been added to the manuscript.

Line 92: Please give more information on chute

Au Response: We do not have a make or model to describe the cattle chutes used. We used four chutes at a time and two were made on the research station. We respectfully suggest that further information is not required for the reader.

Line 94: Please see previous note on changing the timeline (-60, -30, etc).

AU Response: The timeline has been changed in the manuscript as per reviewer #2 suggestion.

Line 95: minutes via jugular catheter

AU Response: The manuscript has been amended to include the Reviewer’s suggestion

Line 95: How were the cows' heads restrained?

AU Response: The following sentence has been inserted into the manuscript. “Directly following the 0-minute sample, the cow’s head was restrained by placing the cow’s head into the head bail, fitting a halter to her head, and tying the halter to the right side of the head bail.

Line 100: 12 to 19 mL seems to be a rather large volume to be sprayed up a cow's nose. Was there any drainage after dosing?

AU Response: No there was no drainage from the cow’s nose.
Line 102: Blood collected via syringe and then transferred? Please describe.

AU Response: We have included a description of the blood collection method in the manuscript

Line 107: Were samples assayed in duplicate?

AU Response: We have included in the manuscript that cortisol and oxytocin analysis was completed with duplicate samples.

Line 108: centrifuged at -4 or 4 degrees C?

AU Response: The blood was centrifuged at 4 degrees Celsius. The manuscript has been amended to state 4 degrees Celsius.

Line 109: until required - for what?

AU Response: The sentence has been amended to state, “All blood tubes were centrifuged at 3,000 × g at 4°C for 15 minutes, after which plasma was removed with a disposable pipette and stored at -20°C until required for analysis.”

Line 122: Day and time of day must be added to the model - if only to prove they had no effect on the model.

AU Response: We did test the model for the effects of day and time (am or pm) and they were not significant. We have included the following sentence in the manuscript, “Day and time of day (AM or PM) were included in the model as fixed effects; however, no effect of either day or time of day was detected and these variables were removed from the model.”

Line 132: What is meant by 'fractious' behavior?

AU Response: We have changed the word ‘fractious’ to the word “uncooperative”

Line 138: remove 'either'

AU Response: The legend in Figure 1. Has been amended by removing the word “either”
Line 161: reduced rather than lesser

AU Response: We have amended the manuscript as suggested by the reviewer

Discussion: Overall the discussion is repetitive and could be reduced significantly. Considering the limited data, and combined Results and Discussion section may be more appropriate.

AU Response: We have eliminated parts of the discussion that was repetitive. The discussion is much shorter in length compared with the discussion length of the original submission

figures - Please add standard error bars to both graphs

AU Response: We provide a measure of variation in the legend of Figure 1 and 2 stating the pooled standard error of the mean. In Figure 3 we present the data for the back transformed mean values for oxytocin. It is incorrect to back transform a measure of variance.

Figure 2: is the spike in lactating oxytocin cows accurate? Observed in all three cows or was there an outlier? This deserves discussion.

AU Response: Yes, the values are correct for the 8 minute time point for the lactating cows treated with intranasal oxytocin (dark circles). The raw values for three cows were 66, 79 and 23.5 the last value (23.5) was the maximum concentration of oxytocin for that animal. The mean value displayed is the back transformed values and are slightly less than the mean of the raw values. The pharmacokinetics of intranasal oxytocin in cattle has been published by Wagner et al. (2020) in which the dose rate used in this study (0.66 IU/kg) had a time to maximum concentration of 4.3 ± 2 mins (Wagner et al. 2020. Short communication: pharmacokinetics of oxytocin administered intranasally to beef cattle. Domestic Animal Endocrinology, 71, <https://doi.org/10.1016/j.domaniend.2019.106387>). It was noted in the Wagner et al. (2020) study that individual variation in time to maximum concentration and maximum concentration reached may have been affected because of thicker mucous or different mucociliary clearance rates affecting the pharmacokinetics and half-life of intranasally supplemented compounds.
